# Some Considerations in the Treatment of the Diseases of Women

**Published:** 1898-12

**Authors:** P. Halsted Fairchild

**Affiliations:** New York


For Texas Medical Journal.

### Some Considerations in the Treatment of the Diseases of Women.

BY P. HALSTED FAIRCHILD, M. D., NEW YORK.

No special field in medicine has been cultivated more assiduously than that of gynaecology. Our journals teem with gynaecological contributions, yet the vast majority of these deal with the surgical aspect of this subject, in which the general practitioner can have but little interest. If one were to judge from the literature, indeed, it would seem that there is no longer such a thing as medical gynaecology, and it is this predominance of operative methods in the management of diseases of woman, which must serve as my apology for venturing to make a few remarks on the medical treatment of these cases.

In view of the fact that the practitioner has an opportunity of treating many of these patients before they come under the observation of the specialist, it depends often upon the thoroughness of his treatment whether resort to the knife will afterwards be necessary. Let us take a familiar instance. A woman consults her physician for a troublesome vaginal discharge. If he looks upon it as a trivial ailment, considers it unnecessary to make an examination, prescribes an astringent injection, what may be the consequences? Endometriatis, salpingitis, pelvic peritonitis may originate in this manner. On the other hand, if he realizes that a failure to determine the exact condition may be attended with the most serious results, his treatment may prevent the extension of the morbid process to the uterus, tubes and ovaries. In conformity with the teachings of some modern authorities, it seems fair to me to assume that in the majority of these cases there is an element of infection which cannot be safely disregarded. Frequently, perhaps usually, the infection is of gonorrhœal character. In fact, as most general practitioners, the writer included, are not in the habit of making examinations for gonococci, it is advisable to treat these patients as if gonorrhœal infection were actually present. Of course, by this it is not to be inferred that there are not many cases of non-gonorrhœal character, but such a line of treatment should be adopted only as a precautionary measure.

It is an important and interesting problem, which yet awaits a satisfactory solution, whether in most instances the infection has not already invaded the uterus before the patient presents herself for treatment. As a rule, this question must be answered in the affirmative from the gynaecologist's standpoint. On the other hand, the family physician who often has an opportunity of seeing the case at a much earlier period is less likely to meet with uterine development. All of which serves to emphasize the statement made above that he is often in a position by timely and well directed treatment to prevent much serious diseases of the uterus and appendages.

A case in point may be of interest. Some years ago the writer was consulted by a married man who during his wife's absence in the country had contracted gonorrhœa, his first attack. On close questioning he confessed, with much reluctance, that since his wife's return he had had connection with her and that he feared she was also suffering from the disease. In extenuation of his brutal conduct he said at the time he cohabited with his wife he had no evidences of gonorrhœa, except an itching about the glans penis and a slight moisture at the meatus. It would therefore appear that he infected his wife at the very beginning of his attack, which at the time of his first visit to me was of one week's duration. The following day his wife came to me for treatment, and examination disclosed a typical gonorrhœal vaginitis and urethritis, the mucous membrane of the vagina being red, swollen and eroded and covered with a foul yellowish creamy secretion. A specimen of the latter was examined microscopically and found to contain gonococci, thus confirming the diagnosis.

What complicated matters considerably in the treatment of this case was that the patient also had some prolapse of the uterus which, however, had never given her any trouble. Having impressed upon her the importance of strictly following the directions she was told to take as much rest as possible and to flush out the vagina morning and night with two or three gallons of warm water by means of a fountain syringe. A nitrate of silver solution, four per cent, was thoroughly applied to the vulva and vagina with a cotton swab, and as the patient was able to report only at long intervals, she was ordered a box of Micajah's Medicated Uterine Wafers, and told to introduce a wafer as far up the vagina as possible after irrigation.

At her next visit—more than a week after—I was agreeably surprised to find that the patient's condition had much changed for the better. The evidences of the inflammatory process were rapidly subsiding; the discharge was much less profuse, thinner and less purulent. The treatment was therefore continued and a permanent cure obtained at the end of four weeks. Although several years have elapsed since then, the patient has not manifested the slightest evidences of uterine disease.

This must be attributed in great part to the promptness with which the treatment was initiated. Had not attention been directed to the undoubted gonorrhœal character of her disease by the husband's confession, and thus an opportunity afforded me of treating the case in its early stage, it is probable that the woman would have waited before consulting a physician until such a time when the infective process had already invaded the uterus, tubes and peritoneum.

In the treatment of gonorrhœal vaginitis, I place great faith in copious vaginal irrigation. No less than two gallons of water, and better still three or four, should be used at each sitting. The temperature of the water should be slightly over 100 degrees F. at the commencement of treatment, but later be increased to a degree compatible with the patient's comfort. It is of great importance that in taking the injections the patient should lie flat on the back with the legs drawn up against the abdomen and the hips slightly raised by means of a pillow. A rubber sheet spread beneath her, with the lower end gathered up in a fold and placed in a pail will prevent the couch or bed from becoming wet.

After the irrigation, it is now my custom to direct the patient to introduce a Micajah Medicated Uterine Wafer as high up in the vagina as possible. Formerly it was my habit to prescribe powders of alum and borax or zinc sulphate, to be added to the water of the douche, but since using the wafers I find that much better results can be obtained more conveniently and agreeably. The wafers have marked astringent and antiseptic properties, and yet are perfectly unirritating. They therefore assist the action of the hot douche in contracting the vessels of the vaginal mucous membrane, relieving the engorgement, promoting the absorption of exudates, and arresting the secretion, while by reason of their powerful antiseptic qualities they destroy the cause of the inflammatory process—the gonococcus. In view of the fact that the wafers dissolve slowly, their action is exerted for a prolonged period and is never marked by an undesirable degree of intensity.

There are two other conditions which are often seen by the general practitioner at a time when by resort to appropriate treatment serious and far-reaching consequences requiring the services of the specialist may be avoided. I refer to perineal lacerations and the so-called "ulceration of the uterine neck." Lacerations of the perineum after childbirth are so common that they do not receive as much consideration as they deserve. Most obstetricians are agreed that if the tear is not too deep and extensive, it is of great advantage to suture it right after childbirth. This has been my custom, and I have rarely found a patient who would object to it. At that time the sensibility of the parts is much reduced, and the sutures can be inserted with but slight pain. Union takes place promptly, as a rule, if care is taken to bring the edges of the tear in accurate apposition. Even small lacerations, however, which require no suturing, demand careful attention, for in these cases there is always a chance that the wound may become infected, and these give rise to mischief. To promote granulation, it is my practice to dust the laceration with boric acid, and insert a uterine wafer into the vagina. In this way infection is prevented, and cicatrization occurs more promptly and completely.

Laceration of the cervix uteri is another condition that the practitioner often has an opportunity of observing and treating at a period before the more of less serious sequelæ have had time to develop. Of course, I am not referring now to extensive tears, which should be repaired by operation as soon as possible, but to the small lacerations which are commonly met with after childbirth. I do not think that a physician is doing his full duty to the patient if he neglects to inspect the os during the early puerperal period. If a laceration be found it should be covered with some antiseptic dusting powder to prevent it from becoming infected. The infection starting in such lacerated cervix tends, in the coure of time, to involve the mucous membrane of the uterus and the uterine appendages. It is therefore advisable, in these cases, to keep the laceration as aseptic as possible, and to encourage granulation. For this purpose the uterine wafers referred to have served me well, by reason of their antiseptic and cicatrizant qualities. One of these wafers should be pushed up against the cervix every day or every other day, and I have found their use extremely cleanly and convenient when compared with a tampon. The same treatment in connection with applications of iodine, and glycerine or ichthyol, proved serviceable in the so-called ulceration of the neck of the womb, a condition in which the cervical mucous membrane is red, swollen, thickened and everted. While a radical cure without operation is usually impossible in these cases, the wafers have an undoubtedly beneficial influence by relieving the congestion and inflammation.

It is not my intention here to discuss the treatment of endometritis, but simply to point out that in these cases also the practitioner sometimes has the advantage of encountering the disease before it has invaded the tubes and the peritoneum, while the specialist usually sees the patient when such invasion has already occurred. Prompt and energetic treatment should therefore be the watchword. Much can be accomplished by measures which will deplete the uterus, relieve the congestion, and establish more normal circulatory conditions. Copious vaginal douching, as described above, is a procedure of great value; the systematic use of the wafers already referred to is no less important. It must be remembered, however, that in many cases we have to deal with an infective process commonly of gonorrhœal character, and that the objects of treatment here are to destroy the germs of infection and then remove the lesions produced by their presence in the mucous membrane. To accomplish this, it may be necessary to resort to intrauterine procedure which the practitioner is unwilling to undertake, and in that event the case should be referred forthwith to the specialist, as time is an element of the utmost importance in the prognosis, and any prolonged delay will materially impair the chances of a permanent cure without serious operative intervention at a subsequent period.

